# Conceptual and empirical problems with game theoretic approaches to language evolution

**DOI:** 10.3389/fpsyg.2014.00226

**Published:** 2014-03-18

**Authors:** Jeffrey Watumull, Marc D. Hauser

**Affiliations:** ^1^Department of Theoretical and Applied Linguistics, University of CambridgeCambridge, UK; ^2^Risk-Eraser, West FalmouthMA, USA

**Keywords:** language evolution, evolutionary game theory, communication, universal grammar, models, theoretical

## Abstract

The importance of game theoretic models to evolutionary theory has been in formulating elegant equations that specify the strategies to be played and the conditions to be satisfied for particular traits to evolve. These models, in conjunction with experimental tests of their predictions, have successfully described and explained the costs and benefits of varying strategies and the dynamics for establishing equilibria in a number of evolutionary scenarios, including especially cooperation, mating, and aggression. Over the past decade or so, game theory has been applied to model the evolution of language. In contrast to the aforementioned scenarios, however, we argue that these models are problematic due to conceptual confusions and empirical difficiences. In particular, these models conflate the comptutations and representations of our language faculty (mechanism) with its utility in communication (function); model languages as having different fitness functions for which there is no evidence; depend on assumptions for the starting state of the system, thereby begging the question of how these systems evolved; and to date, have generated no empirical studies at all. Game theoretic models of language evolution have therefore failed to advance how or why language evolved, or why it has the particular representations and computations that it does. We conclude with some brief suggestions for how this situation might be ameliorated, enabling this important theoretical tool to make substantive empirical contributions.

## INTRODUCTION

Modeling has played a fundamental role in evolutionary biology, including the development of simple yet elegant equations that make predictions about the conditions for the evolution of altruism (Hamilton’s equation: C < rB, C = cost of the behavior, r = degree of genetic relatedness, B = benefit of the behavior) to more complicated equations that explore the dynamics of different selection pressures in the context of different population sizes and migration rates. A fundamental approach for modeling the evolution of behavior is the game theoretic perspective developed by Maynard Smith (1982). Borrowing from economics, Maynard Smith readily saw that a number of significant interactions in animals, including fighting, mating, and predator–prey interactions could be modeled as games, clarifying the costs and benefits of different strategies, and the time course for establishing equilibrium, among other issues. This approach not only helped clarify many important conceptual problems, but helped with the design of empirical experiments centered on these behaviors. Over the past three decades, game theoretic models have made fundamental contributions to our understanding of cooperation in humans and other animals, with models closely tied to empirical tests (Doebeli and Hauert, 2005). Game theoretic models have also been developed to understand the evolution of language. But, we argue, these models have not contributed to conceptual clarity and nor have they been tied to any empirical tests. As such, they have not illuminated our understanding of how language evolved or why it has the particular representational and computational structure that it does. This state of affairs can, however, be remedied, a point that we return to in our conclusion.

## GAME THEORETIC MODELS

By “evolution of language” or “language evolution” we mean the biological evolution of the neurocognitive system of linguistic competence – the system of representations and computations that enables our syntax, semantics, phonology, and the interfaces between them. Given the limitations of comparative research and the paucity of evidence from paleoarcheology and genetics for this system, it would seem that game theoretic modeling could be one of the best (or only viable) approaches to understanding the evolution of language. As a formal system of rules and representations, language could be formalized and understood within a more general abstract model of evolution. The merits of such a model would be many: the explicitness and manipulability of conditions and relations generating consequences to exploring the robustness and empirical plausibility of the modeled system. With such clarification, researchers could then investigate how closely nature actually approximates these idealizations. Indeed it was the expectation of such benefits in the domain of population genetics that motivated Lewontin (1961) to build evolutionary game theory on the mathematical foundations established by Fisher (1930), von Neumann and Morgenstern (1944), among others. As Lewontin noted, the behaviors of idealized agents – humans and nonhumans (e.g., animals, bacteria, plants, genes, etc.) – engaged in competition are governed by or measured against general rational principles that necessarily determine winning/losing strategies in specific idealized circumstances. The classic models identified rigorously formulable and empirically verifiable stable strategies approximated by different organisms. This contrasts with modern models, which simulate the “dynamic” evolution of populations of strategies rather than strategy-using individuals.

## DEFINITIONS AND DISTINCTIONS

Dynamic computational simulations generate insights insofar as the simulated system is rigorously defined in an abstract mathematical theory. For instance, the important game theoretic simulations on the evolution of cooperation run by Axelrod and Hamilton (1981) were based on mathematically idealized scenarios of animal conflict (Maynard Smith and Price, 1973) and reciprocation (Hamilton, 1964; Trivers, 1971). Such mathematical foundations are nonexistent in computational models of the evolution of language for the simple reason that most of language has yet to be sufficiently idealized and mathematized. For those aspects of language that have been adequately formalized, game theoretic models of syntax (Ristad, 1993) and genetic algorithms as acquisition mechanisms (Clark and Roberts, 1993) have been successful in explaining properties of the *design* of the language faculty, with no pretense to explain its *use* in behaviors. This, we submit, is the correct approach logically, theoretically, and methodologically. Logically, identifying the *mechanism*, even if not understood completely, must precede and direct investigation of its *functions*. Theoretically, if we are interested in the *biological* basis of linguistic competence, language needs to be modeled as a property of individuals, which are subject to *biological* evolution, not as a set of strategies, which are not. Moreover, as some have argued, the use of language is ancillary to its design (Chomsky, 2013). Methodologically, there are virtually infinitely many uses of language so that zooming in on one is in some sense arbitrary and zooming out to all is impractical. And of course as a mechanism evolves, its uses can change (Gould and Vrba, 1982). Nevertheless evolutionary simulations of language have all but exclusively modeled use. In particular, these models assume that the language phenotype emerges in the dynamics of communication. This assumption, we suggest, is unfounded.

## LANGUAGE AND COMMUNICATION

Language cannot, and should not be reduced to communication. Even if it could, however, the computational model would be vacuous pending a mathematical theory of communication. Shannon’s (1948) mathematical theory of communication is a start, but the explanatory power and formal beauty of that theory is in its abstraction from meaning so as to define and quantify information. This information theoretic notion of communication is obviously important to – though obviously far from sufficient for – understanding linguistic communication, and it is not the notion assumed in evolutionary simulations of language. In these models, the transmission of meaning is the raison d’être of communication; game theoretically, agents receive positive payoffs for successful communication of referents (Nowak and Komarova, 2001). This is problematic because there is no mathematical theory of communication in this sense to be modeled; nor is this sense sufficient to encompass our communicative use of language, which is boundless: “We inform, we request, we persuade, we interrogate, we orate, and sometimes we just schmooze” (Pinker, 2007). There are simply too many strategies to model: agents communicate to trade knowhow (Pinker and Bloom, 1990), deceive (Dawkins and Krebs, 1978), negotiate social relations (Dunbar, 1996), to list but a few. Many such uses surely were adaptive, although establishing this empirically for our hominin ancestors is surely impossible, for we cannot travel back to listen (or look) in. Computer models of these strategies show how they could have proliferated, but whether this corresponds to reality is unknown and probably unknowable for the simple reason that there are no viable empirical tests. Furthermore, and fundamentally, none of these uses explains the emergence of the system to be so used. In short, it may be that “computer models are good for demonstrations of how different uses of language provide benefit – but they are not much good for proving why and how language evolved” (Livingstone, 2003).

A game theoretic model – verified experimentally – can, for instance, successfully demonstrate the costs/benefits of particular pragmatic acts (Lee and Pinker, 2010), but nothing so theoretically rigorous and empirically robust has been formulated for language in general, and in particular, for the underlying competence of representations and computations. Importantly, we do not know how canalized (genetically, epigenetically, anatomically) the evolution of this machinery was, so that game theory – which assumes a sizable set of possibilities – may be largely inapplicable. This fact has prevented modeling attempts to explain the evolution of language.

## FLAWS IN THE STANDARD GAME THEORY MODEL OF LANGUAGE EVOLUTION

A standard game theoretic model of language (Nowak and Komarova, 2001; Nowak et al., 2002) is formatted as a set of matrices. The first “competence” matrix defines a language *L *as a set of sound-meaning associations. This is already questionable because linguistic competence is not a set of structures but rather the mechanism generative of such structures (Watumull, 2012). Two more matrices, *P* and *Q*, represent “performance,” with *P* the set of probabilities that a speaker of *L*_I_ uses sound *j* to communicate meaning *i*, and *Q* the set of probabilities that a hearer with knowledge of L*_J_* (not necessarily distinct from *L*_I_) understands sound *j* as meaning *i*. Needless to say, the specification of these probabilities is arbitrary (and biases the outcomes) and empirically empty absent any testable mapping to reality. It is also questionable to stipulate the fidelity of transmission of meaning as *the* purpose of communication and language (the metric of its “success”) for there are as many models – many of which are formally and empirically solid (Pinker et al., 2008) – showing ambiguity to be a successful communicative strategy built into our pragmatic competence. And, to reiterate, the equation of communication with language is a conflation of function with mechanism: playing any of these games presupposes the linguistic machinery to be explained in the evolutionary theory.

Returning to the standard model, σ is defined (over all possible meanings) as the probability that sound *j* does in fact communicate meaning *i*. Again, no empirical basis is given for specifying one probability rather than another; different probabilities yield different but equally (im)plausible – hence equally (un)interesting – results. The final probability that a speaker of *L_I_* produces a sound *j* decoded with meaning *i* by hearer with *L_J_* is defined a*_IJ_* =∑*_ij_* σp*_ij_* q*_ij_* . From this foundation, the game theoretic payoff between *L_I_* and *L_J_* is defined as F*_IJ_* =1/2(*a_IJ_ +a_JI_*). The “communicative payoff” of *L_I_* is thus *F_II_* = *a_II_*. The communicative payoff is a number *k* between 0 and 1, *k* < 1 if the signal is ambiguous or in some way impoverished. The higher the communicative payoff of a language, the higher its “fitness” in that a language is transmitted generation to generation in proportion to its communicative payoff. But, since fitness applies to biological entities, not sound-meaning pairs, this methodological approach is highly questionable. In these models, more offspring are born to parents with more successful languages. However, this presupposes the evolution of an acquisition device enabling sophisticated imitation, which we know is unique to humans among primates (Petkov and Jarvis, 2012). So once again, the models *might* demonstrate how systems proliferate, but not how they emerged – the fundamental question of language evolution.

Finally, following Chomsky (1965) and Gold (1967), some of these models do accept the need to explain the evolution of the genetic components of grammar, but mistake “the crucial question” to be “what makes a population of speakers converge to a coherent grammatical system. In other words, what are the conditions (grammar) has to fulfill for a *population* of individuals to evolve coherent communication?” (Nowak and Komarova, 2001). These are the wrong questions for modeling the evolution of the language faculty. Rather, we must ask what emerged in the brain to enable the representational and computational bases of language? This question is distinct from that of the conditions this system had to satisfy to be learnable, usable, and ultimately, genetically fixated in a population. Communication was probably very important in the fixation process, but as with work on animal communication (Hauser, 1996), we need extensive empirical studies to even approach understanding.

## CONCLUSION

In constrast to the substantial contribution of evolutionary game theory to understanding cooperation, mating behavior, and aggression, its contribution to understanding language evolution has been substantially less. This is due to its (1) failure to distinguish between the mechanisms that constitute language and how such mechanisms enable communicative expression; and its (2) inability to generate a single empirical study to either support or reject its predictions or assumptions. Given that we lack organisms or situations in which such tests could be carried out, we suggest that it will be exceedingly difficult for game theory to advance our understanding of how language evolved, why it was designed with its particular representations and computations, and what selection pressures led to such design features. We cannot endorse the proliferation of game theory models to understand the evolution of language until a clear empirical agenda is established, and until the target of inquiry is the evolution of our biological capacity for language as opposed to one of its functions in communication. In this light, we suggest that future attempts to model language evolution from a game theoretic perspective focus on understanding its computations and representations, including why some formats or generative procedures might win out over others, and how different language acquisition devices might compete, with the goal of attaining optimal competences in the knowledge acquired and expressed. Though empirical verification of such models will be challenging, once the right models are created, the different strategies may well be deployed in artificial agents via simulation. These results would at least add to the plausibility of the theoretical predictions of game theory modeling.

## Conflict of Interest Statement

The authors declare that the research was conducted in the absence of any commercial or financial relationships that could be construed as a potential conflict of interest.
